# Systemic immune-inflammation index within the first postoperative hour as a predictor of severe postoperative complications in upper abdominal surgery: a retrospective single-center study

**DOI:** 10.1186/s12876-022-02482-9

**Published:** 2022-08-27

**Authors:** Yang Jiao, Xiao Zhang, Mei Liu, Yu’e Sun, Zhengliang Ma, Xiaoping Gu, Wei Gu, Wei Zhu

**Affiliations:** Department of Anesthesiology, Nanjing Drum Tower Hospital, The Affliated Hospital of Nanjing University Medical School, 321 Zhongshan Road, Nanjing, 210008 China

**Keywords:** Systemic immune-inflammation index, Upper abdominal surgery, Severe postoperative complications

## Abstract

**Background:**

Systemic pro-inflammatory factors play a critical role in mediating severe postoperative complications (SPCs) in upper abdominal surgery (UAS). The systemic immune-inflammation index (SII) has been identified as a new inflammatory marker in many occasions. The present study aims to determine the association between SII and the occurrence of SPCs after UAS.

**Methods:**

Included in this study were 310 patients with upper abdominal tumors who received UAS and subsequently were transferred to the anesthesia intensive care unit between November 2020 and November 2021 in Nanjing Drum Hospital. SPCs, including postoperative pulmonary complications (PPCs), major adverse cardiac and cardiovascular events, postoperative infections and delirium, were recorded during the hospital stay. The clinical features of the patients with and without SPCs were compared by Student’s t-test or Fisher’s exact test as appropriate. Risk factors associated with SPC occurrence were evaluated by univariable and multivariable logistic regression analyses. Receiver operating characteristic (ROC) curve analysis was used to establish a cut-off level of SII value to predict SPCs.

**Results:**

Of the 310 patients receiving UAS, 103 patients (33.2%) presented at least one SPC, including PPCs (n = 62), adverse cardiovascular events (n = 22), postoperative infections (n = 51), and delirium (n = 5). Both preoperative SII and 1-h postoperative SII in patients with SPCs were significantly higher than those in patients without SPCs. Multivariate analysis showed that 1-h postoperative SII was an independent predictor for SPC occurrence (OR = 1.000, 95% CI 1.000–1.000, *P* = 0.007), together with postoperative C-reactive protein, postoperative arterial lactate, postoperative oxygenation-index and older age. The ROC curve showed that the optimal cutoff value of 1-h postoperative SII to predict SPCs was 754.6078 × 10^9^/L, with an 88.3% sensitivity and a 29% specificity. Multivariate analysis also confirmed that 1-h postoperative SII > 754.6078 × 10^9^/L was associated with increased SPC occurrence (OR = 2.656, 95% CI 1.311–5.381, *P* = 0.007).

**Conclusion:**

Our findings demonstrated an association between the higher level of 1-h postoperative SII and SPCs, suggesting that 1-h postoperative SII, especially categorized 1-h postoperative SII using cutoff value, may be a useful tool for identifying patients at risk of developing SPCs.

## Introduction

Postoperative complications, especially Clavien-Dindo III-V complications [1], heavily contribute to the high mortality of patients receiving upper abdominal surgery (UAS) [2]. Severe postoperative complications (SPCs), including postoperative pulmonary complications (PPCs), cardiovascular events, infections and delirium, are commonly seen after upper abdominal surgeries, causing poor outcomes [3, 4]. In addition, these SPCs also prolong the time of hospitalization and increase the economic burden. Early recognition and timely intervention to avoid the progression of SPCs help improve the prognosis of the patients [5, 6]. Therefore, it is necessary to find proper indicators to identify patients at risk of developing SPCs.

Some perioperative inflammation markers such as high-sensitivity complement reactive protein (CRP) and procalcitonin (PCT) have been established as risk predictors to estimate the risk of SPC occurrence [7, 8]. However, the high cost of detecting these markers and strict restrictions on the test conditions limit their wider use as clinical routine tests, especially in some developing countries. Inflammatory indicators derived from different combinations of neutrophils, lymphocytes and platelets such as the neutrophil to lymphocyte ratio (NLR) and the platelet to lymphocyte ratio (PLR) have been demonstrated as useful prognostic tools in many solid malignant tumors [9, 10]. Recently, a novel parameter known as "systemic immune-inflammation index" (SII: platelet count × neutrophil count/lymphocyte count) has been shown to be superior to PLR and NLR in that it can better reflect the outcome differences in both operation and nonoperation settings [11, 12]. The postoperative complications generally are affected by the host inflammatory and immune response status, suggesting that SII may be also a useful tool to stratify patients at risk of developing SPCs after UAS.

The aim of the present study was to assess the distribution of SPCs in patients with upper abdominal tumors who underwent UAS in our center, and explore the prognostic value of SII in predicting the postoperative occurrence of SPCs.

## Patients and methods

### Study design and participants

**Fig. 1:**
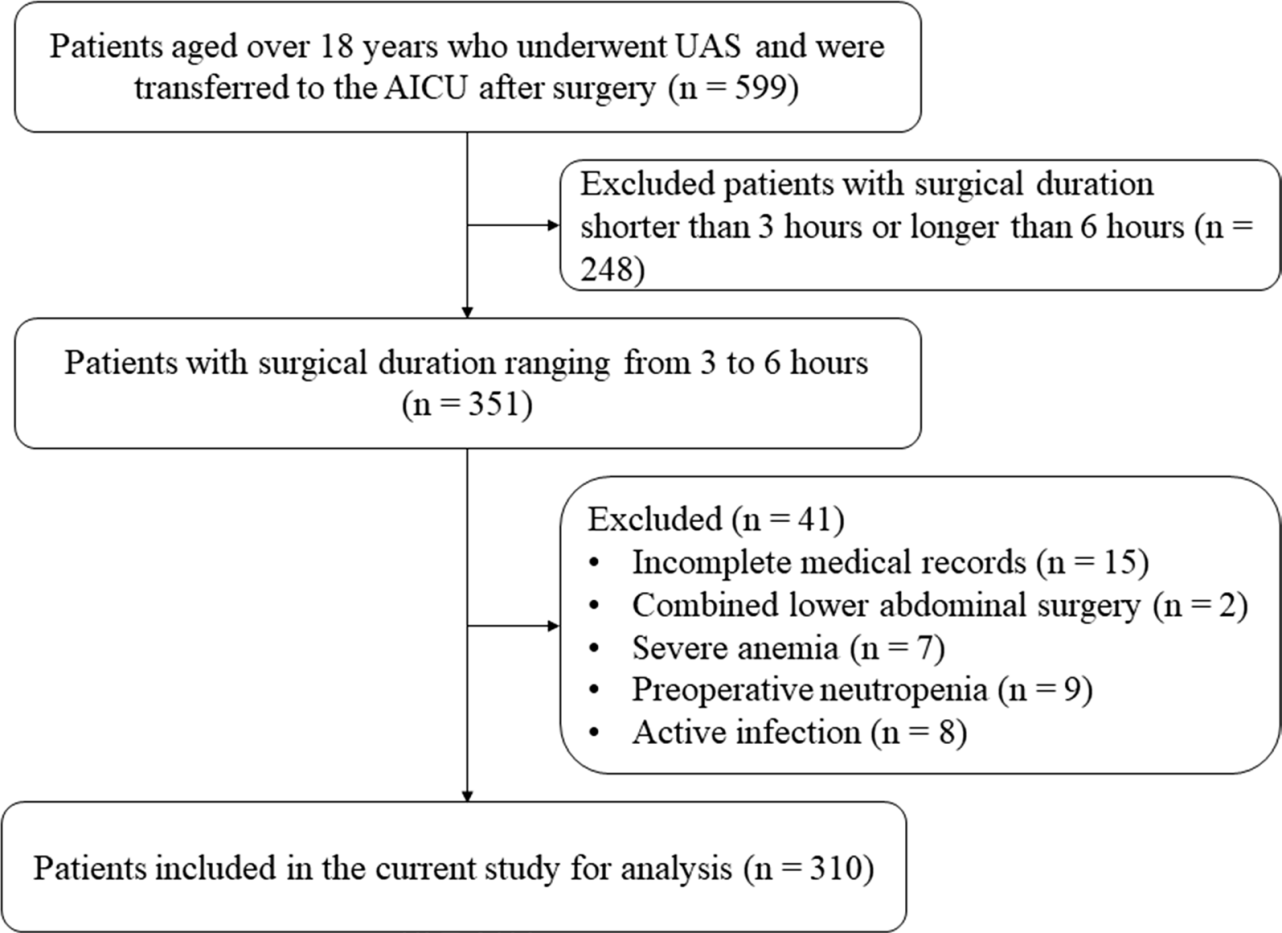
The flow chart diagram for the patient enrollment. UAS, upper abdominal surgery; AICU, anesthesia intensive care unit

As a practice in our center, patients at risk of delayed extubation (unstable haemodynamics, high airway pressure, hyoxemia, large amount of muscle relaxant administration, surgical duration ≥ 3 h) after surgery would be transferred to the anesthesia intensive care unit (AICU) for further observation. Included in this study were AICU patients who received UAS and subsequently transferred to the AICU of Nanjing Drum Hospital (Nanjing, China) between November 2020 and November 2021. The inclusion criteria were (1) patients aged over 18 years who were diagnosed with upper abdominal tumors and received UAS including the Whipple procedure, liver resection, cholecystectomy, bile duct resection or gastrectomy; (2) the surgical duration ranging from 3 to 6 h; and (3) patients who were transferred to the AICU after surgery. The exclusion criteria were patients with incomplete medical records, who underwent complex surgical procedures, with severe anemia (preoperative hemoglobin < 70 g/L), active bleeding, preoperative neutropenia (< 1 × 10^9^/L) and/or active infection (preoperative total leukocyte count > 11 × 10^9^/L). (Fig. 1) Prior approval for this retrospective study was obtained from the institutional ethics committee of said hospital (Approval No. 2021-563-01).

### Data collection

Following data were obtained from the medical records of the patients: age, sex, body mass index (BMI), smoking history, concomitant diseases including hypertension, diabetes mellitus (DM), coronary artery disease (CAD), and chronic obstructive pulmonary disease (COPD). Laboratory findings within three days before operation and within the first postoperative hour after transferring to the AICU were also recorded, including hemoglobin (Hb), total leukocyte count (TLC), differential leukocyte count (DLC), platelet count (PLT), blood urea (BU), serum creatinine (SCR), albumin, aspartate-transaminase (AST) and alanine-transaminase (ALT) levels, C-reactive protein (CRP), oxygen index (PaO_2_/FiO_2_), neutrophil–lymphocyte ratio (NLR), platelet-lymphocyte ratio (PLR), and SII (platelet count × neutrophil count/lymphocyte count). Perioperative parameters including the operation type and duration were also recorded.

### Definitions

The complications were studied according to the Clavien-Dindo scale [1]. PPCs, major adverse cardiac and cardiovascular events, postoperative infections, and delirium were defined as SPCs. The primary outcome was the incidence of the presence of ≥ 1 SPC in patients who underwent UAS. The diagnosis with pneumonia, tracheobronchitis, atelectasis with clinical repercussion, acute respiratory failure, bronchoconstriction was regarded as PPC. Major adverse cardiac and cardiovascular events were characterized by any of the following: ST-segment elevation myocardial ischemia, cardiac failure, cardiac arrest, cerebrovascular accident, and/or transient ischemic attack. Postoperative infectious complications were defined according to the European Society of Anaesthesiology-European Society of Intensive Care Medicine guidelines of 2015 as surgical site infection, pneumonia, bloodstream infection, urinary tract infection and infection of unknown sources [13]. Delirium was diagnosed based on the criteria in the fifth edition of the Diagnostic and Statistical Manual of Mental Disorders [14].

### Statistical analysis

The normal distribution of the continuous variables was tested using the Shapiro–Wilk test. Normally distributed data are presented as the mean ± standard deviation (SD). Comparisons between two groups under identical conditions were performed by the 2-tailed Student’s t test. Non-normally distributed data are presented as the median (25th–75th percentile). Comparisons between two groups were performed by Mann–Whitney U test. The categorical variables were expressed as number of patients and percentage of patients and compared between the groups using the chi-square test. Univariate logistic regression was performed to identify associations between postoperative complications and other variables. Multivariable logistic regression was performed using variables with a *P* value < 0.05 in the univariate analysis. Using a receiver operating characteristic (ROC) curve, we explored the predictive value of variables included in the multiple logistic regression for poor outcomes. The prediction model was evaluated by the area under the curve (AUC). The “optimum cutoff point” was determined using Youden index, as the cutoff point with the highest (sensitivity + specificity − 1), at which there was a maximal correct classification of the unfavorable outcomes. The statistical software SPSS version 22 (IBM Corp, Armonk, NY) was used for the analysis. All tests were two-sided, and a *P* value < 0.05 was considered to indicate significance.

## Results

### SPC distribution

Of the 310 patients with upper abdominal tumors enrolled in this study, Whipple procedure was performed in 25 patients, liver resection in 117 patients, cholecystectomy in 10 patients, bile duct resection in 10 patients, and gastrectomy in 148 patients. During the hospitalization period, 103 patients (33.2%) presented at least one SPC, including PPCs, adverse cardiovascular events, postoperative infections or delirium in 62 (20%), 22 (7.1%), 51 (16.5%) and 5 (1.6%) patients respectively. Of the 310 patients, 76 (24.5%) had only one complication, 18 (5.8%) had two, and 9 (2.9%) had three or more than three complications. There was no significant difference in complication occurrence between patients who received different surgeries. The details of complication occurrence are shown in Table 1.

**Table 1:** Severe postoperative complications in patients with upper abdominal tumors who received upper abdominal surgery

	Whipple procedures (n = 25)	Liver resection (n = 117)	Cholecystec-tomy (n = 10)	Bile duct resection (n = 10)	Gastrectomy (n = 148)	*P* value
PPC	5 (20%)	20 (17.1%)	4 (40%)	4 (40%)	29 (19.5%)	0.22
ACE	4 (16%)	8 (6.8%)	0 (0%)	0 (0%)	10 (6.8%)	0.33
Infections	8 (32%)	12 (10.3%)	2 (20%)	3 (30%)	26 (17.6%)	0.06
Delirium	1 (4%)	0 (0%)	0 (0%)	0 (0%)	4 (2.7%)	0.37
With 1 complication	5 (20%)	29 (24.8%)	5 (50%)	5 (50%)	32 (21.6%)	0.09
With 2 complications	2 (8%)	7 (6.0%)	0 (0%)	1 (10%)	8 (5.4%)	0.87
≥ with 3 complications	2 (8%)	2 (1.7%)	0 (0%)	0 (0%)	4 (2.7%)	0.43

Data are presented as number (%)

*PPC* postoperative pulmonary complication, *ACE* adverse cardiovascular event

### Demographic and clinical characteristics

The mean age of the patients in our series was 63.9 ± 11.6 years, including 215 (69.3%) male patients. The demographic variables, preoperative and 1-h postoperative biochemical tests were compared between patients with or without SPCs. The results showed that patients who developed SPCs were significantly older in age and had higher preoperative levels of SII. Expectedly, patients with complications had a significantly longer duration of ventilation and longer time of ICU stay and hospital stay (Table 2). Notably, the 1-h postoperative biochemical parameters showed that the patients with complications had significantly higher levels of arterial lactate, CRP and SII, and lower level of oxygen index (PaO_2_/FiO_2_) (Table 3). All other baseline characteristics including gender, BMI, ASA (American Society of Anesthesiologists) classification, concomitant diseases, smoking histories and laboratory results were comparable between the two groups (Table 2).

**Table 2:** Baseline characteristics and perioperative characteristics of patients with and without SPCs

Variable	Without SPCs (n = 207)	With SPCs (n = 103)	*P* value
Age (years)	64 (56–71)	68 (60–73)	0.002**
Male sex	145 (70%)	70 (68%)	0.707
ASA classification > 2	112 (54.1%)	59 (57.3%)	0.596
BMI (kg/m^2^)	22.92 (20.66–25.36)	23.00 (21.30–25.63)	0.803
History of smoking	40 (19.3%)	21 (20.4%)	0.824
Hypertension	74 (35.7%)	42 (40.8%)	0.389
DM	30 (14.5%)	18 (17.5%)	0.494
CAD	6 (2.9%)	7 (6.8%)	0.193
COPD	2 (1.0%)	4 (3.9%)	0.187
Preoperative TLC (10^9^/L)	5.20 (4.00–6.30)	5.20 (4.40–6.60)	0.243
Preoperative NLR	2.21 (1.58–3.15)	2.27 (1.61–3.20)	0.508
Preoperative PLR	123.64 (89.96–160.00)	131.43 (95.00–186.43)	0.112
Preoperative SII (10^9^/L)	358.71 (215.36–553.56)	463.95 (269.12–702.65)	0.027*
Preoperative Hb (g/L)	130 (117–142)	128 (114–143)	0.709
Preoperative albumin (g/L)	39.40 (37.70–41.40)	39.00 (36.80–41.60)	0.296
Preoperative AST (U/L)	20.50 (17.00–31.10)	21.40 (17.80–33.40)	0.225
Preoperative ALT (U/L)	20.30 (13.00–31.40)	20.40 (13.10–43.40)	0.606
Preoperative serum creatinine (µmol/L)	65 (54–73)	63 (54–74)	0.721
Preoperative Urea (mmol/L)	5.30 (4.40–6.30)	5.00 (4.10–6.00)	0.111
Preoperative CRP (mg/L)	3.40 (2.20–5.90)	3.30 (2.20–8.10)	0.488
Preoperative APTT (s)	26.90 (25.40–28.70)	26.60 (25.70–28.50)	0.925
Duration of surgery (h)	4.00 (3.30–4.60)	4.00 (3.50–5.00)	0.054
Length of MV (h)	2.50 (1.75–4.00)	3.00 (2.25–4.25)	0.022*
Length of AICU stay (days)	1 (1–1)	1 (1–1)	0.045*
Length of hospital stay (days)	16 (14–20)	21 (16–28)	< 0.001***

*SPCs* Severe postoperative complications, *ASA* American Society of Anesthesiologists, *BMI* body mass index, *DM* diabetes, *CAD* coronary artery disease, *COPD* chronic obstructive pulmonary disease, *TLC* total leukocyte count, *NLR* neutrophil count/lymphocyte count, *PLR* platelet count/lymphocyte count, *SII* platelet count × neutrophil count/lymphocyte count, *Hb* hemoglobin, *AST* aspartate-transaminase, *ALT* alanine-transaminase, *CRP* C-reactive protein, *APTT* activated partial thromboplastin time, *MV* mechanical ventilation, *AICU* anesthesia intensive care unit

*P < 0.05, **P < 0.01, ***P < 0.001

**Table 3:** The 1-h postoperative laboratory parameters of patients with and without SPCs

Variable	Without SPCs (n = 207)	With SPCs (n = 103)	*P* value
Lactate (mmol/L)	0.90 (0.70–1.40)	1.10 (0.80–1.80)	0.004**
Oxygenation-index	442 (368–498)	418 (286–470)	0.002**
TLC (10^9^/L)	8.40 (6.60–11.00)	9.00 (6.70–12.10)	0.102
NLR	9.50 (5.36–18.67)	11.20 (7.36–23.60)	0.042*
PLR	187.50 (133.64–298.00)	245 163.75–350.00)	0.018*
SII (10^9^/L)	1482.00 (724.62–2649.60)	1840.44 (1040.00–3210.00)	0.013*
Hb (g/L)	109 (97–121)	108 (97–122)	0.994
Albumin (g/L)	27.60 (24.20–31.60)	27.40 (23.90–31.00)	0.449
AST (U/L)	67.00 (36.00–160.00)	96.00 (45.50–182.00)	0.073
ALT (U/L)	58 (34–141)	75 (44–158)	0.137
Serum creatinine (µmol/L)	57.20 (46.90–67.80)	58.90 (46.10–70.30)	0.692
Urea (mmol/L)	4.76 (4.00–5.89)	4.91 (3.83–5.85)	0.931
CRP (mg/L)	31.30 (17.80–45.70)	34.40 (24.20–58.40)	0.023*
APTT (s)	29.50 (27.10–32.40)	29.70 (26.80–32.80)	0.694

*SPCs* severe postoperative complications, *TLC* total leukocyte count, *NLR* neutrophil/lymphocyte count, *PLR* platelet /lymphocyte count, *SII* Systemic immune-inflammation index, *Hb* hemoglobin, *AST* aspartate-transaminase, *ALT* alanine-transaminase, *CRP* C-reactive protein, *APTT* activated partial thromboplastin time

*P < 0.05, **P < 0.01, ***P < 0.001

### 1-h postoperative SII is independently associated with complication occurrence

Five statistically significant variables in univariate logistic regression analysis were subjected to multivariate logistic regression. These variables included the age (OR = 1.037, *P* = 0.002), 1-h postoperative arterial lactate (OR = 1.316, *P* = 0.021), 1-h postoperative oxygenation-index (OR = 0.995, *P* < 0.001), 1-h postoperative CRP (OR = 1.011, *P* = 0.014) and 1-h postoperative SII (OR = 1.000, *P* = 0.007). The result of multivariate logistic regression analysis showed that age (OR = 1.032, 95% CI 1.006–1.059), 1-h postoperative arterial lactate (OR = 1.468, 95% CI 1.136–1.898), 1-h postoperative oxygenation-index (OR = 0.995, 95% CI 0.992–0.998), 1-h postoperative CRP (OR = 1.012, 95% CI 1.002–1.022), and 1-h postoperative SII (OR = 1.000, 95% CI 1.000–1.000) remained to be independent predictors of complication occurrence (Table 4). However, the significance of preoperative SII for prediction has not been observed in our study.

**Table 4:** Factors associated with SPCs in UAS patients as determined by univariate and multivariate analyses

Variables	Univariate analysis		Multivariate analysis	
	OR (95% CI)	*P* value	Adjusted OR (95% CI)	*P* value
Age (years)	1.037 (1.014–1.062)	0.002**	1.032 (1.006–1.059)	0.015*
Preoperative SII (10^9^/L)	1.001 (1.000–1.001)	0.058		
Postoperative lactate (mmol/L)	1.316 (1.043–1.661)	0.021*	1.468 (1.136–1.898)	0.003**
Postoperative oxygenation index	0.995 (0.992–0.997)	< 0.001***	0.995 (0.992–0.998)	0.001***
Postoperative NLR	1.013 (0.995–1.032)	0.169		
Postoperative PLR	1.001 (1.000–1.002)	0.062		
Postoperative SII (10^9^/L)	1.000 (1.000–1.000)	0.007**	1.000 (1.000–1.000)	0.007**
Postoperative CRP (mg/L)	1.011 (1.002–1.020)	0.014*	1.012 (1.002–1.022)	0.016*

*SPC* severe postoperative complications, *SII* Systemic immune-inflammation index, *NLR* neutrophil/lymphocyte count, *PLR* platelet/lymphocyte count, *CRP* C-reactive protein

*P < 0.05, **P < 0.01, ***P < 0.001

**Fig. 2:**
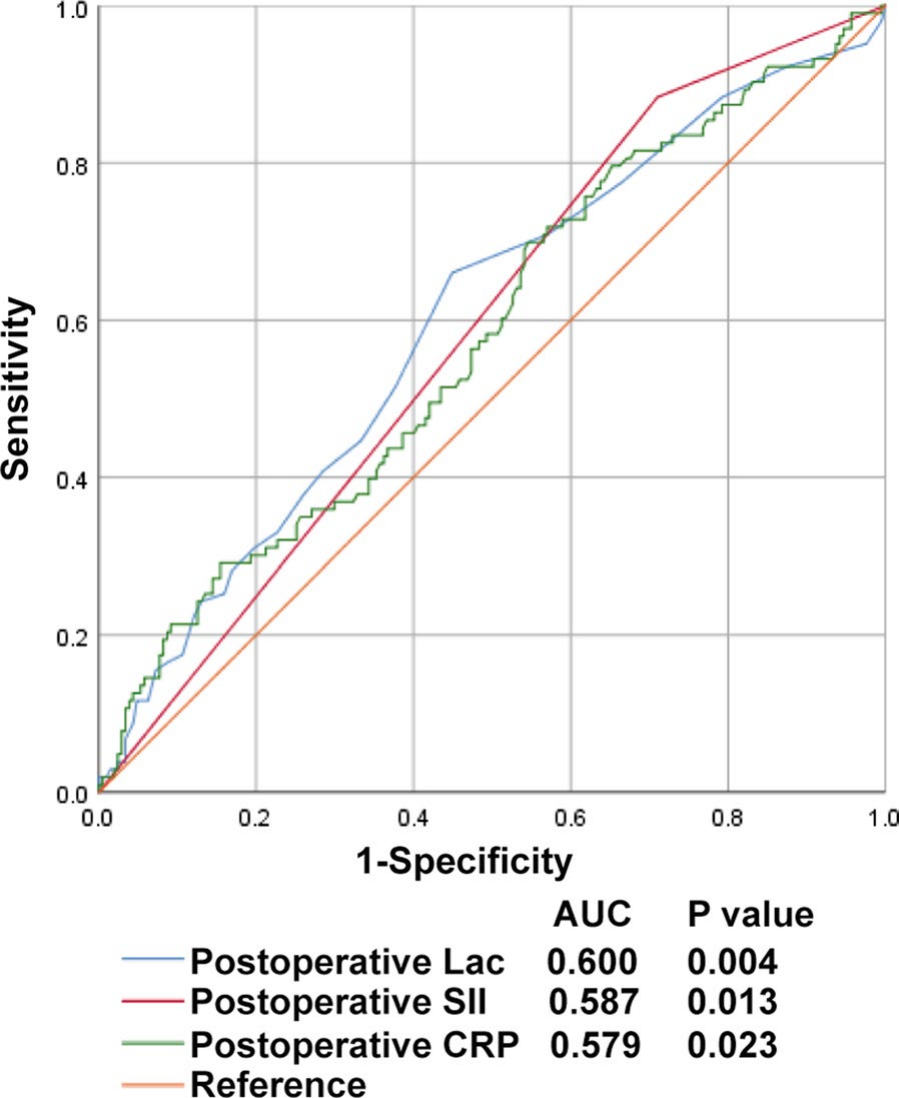
Receiver operator characteristic curve of the individual variables for predicting SPCs. Lac, Lactate; SII, Systemic immune-inflammation index; CRP, C-reactive protein; AUC, area under the receiver operating characteristic curve

**Fig. 3:**
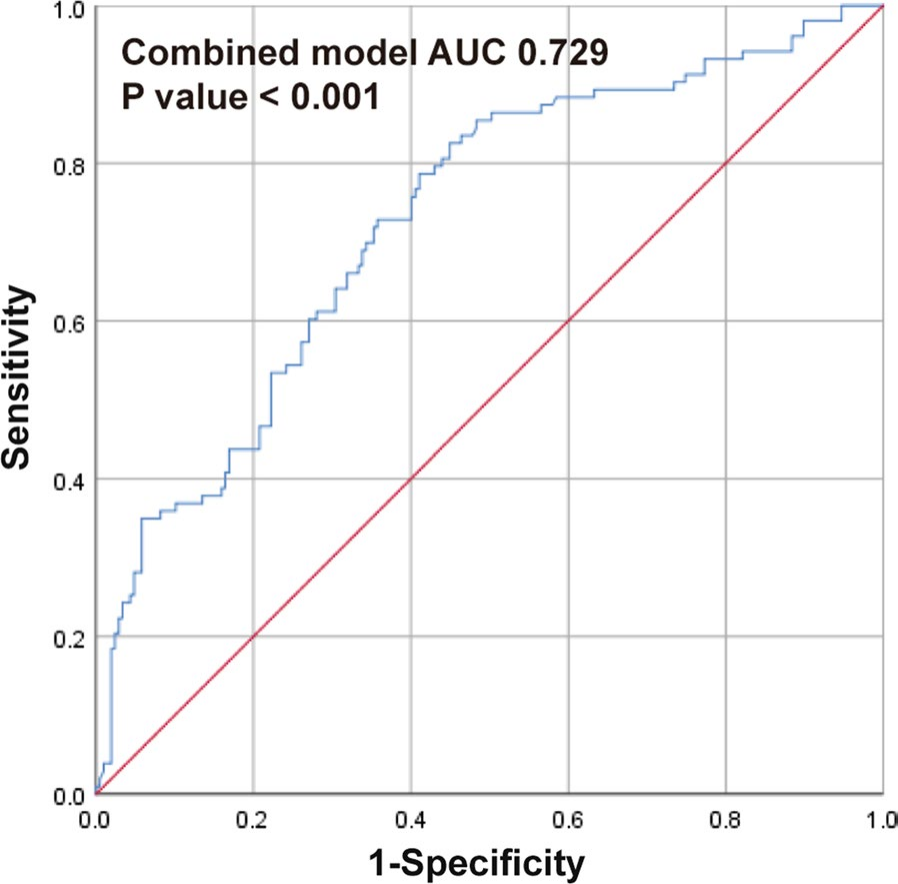
Receiver operator characteristic curve of the combined model for predicting SPCs. AUC, area under the receiver operating characteristic curve

The AUC for 1-h postoperative SII was 0.587 (95% CI, 0.521–0.653; *P* = 0.013), and the best cutoff value of 1-h postoperative SII was 754.6 × 10^9^/L, offering an 88.3% sensitivity and a 29% specificity for prediction (Fig. 2). Based on the cutoff value of 1-h postoperative SII, univariate and multivariate logistic regression analyses were performed again. The result of logistic regression analysis also confirmed that 1-h postoperative SII > 754.6078 × 10^9^/L was associated with increased SPC occurrence (OR = 2.656, *P* = 0.007) (Table 5). Combined with all the independent associated parameters, the AUC for the selected predicting model was 0.716 (95% CI, 0.654–0.778; *P* < 0.001) (Fig. 3).

**Table 5:** Multivariate logistic regression analysis after categorizing 1-h postoperative SII using cutoff value

Variables	Adjusted OR	95% CI	*P* value
Age (years)	1.032	1.007–1.059	0.014*
Postoperative lactate (mmol/L)	1.389	1.076–1.793	0.012*
Postoperative oxygenation-index	0.996	0.993–0.998	0.002**
Categorized postoperative SII (10^9^/L)	2.656	1.311–5.381	0.007**
Postoperative CRP (mg/L)	1.012	1.002–1.022	0.014*

*SII* Systemic immune-inflammation index, *CRP* C-reactive protein

*P < 0.05, **P < 0.01

In addition, the complications between patients with 1-h postoperative SII ≥ 754.6 × 10^9^/L and those with < 754.6 × 10^9^/L were compared. The results showed that patients with SII ≥ 754.6 × 10^9^/L had significantly higher incidence of developing PPCs and postoperative infectious complications than those with 1-h postoperative SII < cutoff value (Table 6).

**Table 6:** Comparison of SPCs between the groups with 1-h postoperative SII ≥ cutoff and SII < cutoff value

Postoperative complications	SII < 754.6 × 10^9^/L (n = 72)	SII ≥ 754.6 × 10^9^/L (n = 238)	*P* value
PPCs	7 (9.7%)	55 (23.1%)	0.013*
Adverse cardiovascular events	2 (2.8%)	20 (8.4%)	0.103
Infectious postoperative complications	6 (8.3%)	45 (18.9%)	0.034*
Delirium	0 (0%)	5 (2.1%)	0.215

*PPCs* postoperative pulmonary complications, *SII* Systemic immune-inflammation index

*P < 0.05

## Discussion

In our study, the composite events including PPCs, cardiovascular complications, infections, and delirium were applied as analytical indexes instead of an isolated one, knowing that systemic inflammation is involved in the whole process of pathogenesis. In addition, our study focused on the clinical and prognostic value of biochemical data obtained within 1-h post-operation. These data may provide the information about the whole-body response after surgery and the potential complications in an early stage. Overall, our results suggest that 1-h postoperative SII is an independent factor for predicting SPC occurrence in UAS patients.

Pulmonary complications and postoperative infectious complications are leading causes of morbidity and mortality following UAS [15]. The incidence of PPCs in this population ranges from 9 to 40%, depending on the criteria used for the diagnosis of PPCs [16]. It was found in our study that the PC occurrence rate was 20%, which is similar to other published reports. Generally, gender, COPD, congestive heart failure, diabetes, age, and surgery are considered as the risk factors associated with PPCs [17, 18]. However, there is no reliable biomarker for predicting PPCs at present. Accumulating evidence has shown that SII plays a predictive role in the diagnosis and prognosis prediction of many lung diseases. Gok et al. [19] showed that SII was a powerful tool for predicting the severity of pulmonary embolism. Watanabe et al. [20] reported that NSCLC patients with elevated SII values had a higher risk of early recurrence. From a micro-view, local lung inflammation triggers a systemic response, manifested as increased neutrophil and thrombocyte counts and a decreased lymphocyte count. Changes in neutrophil, thrombocyte and lymphocyte counts generally are earlier than the onset of PPCs or infection symptoms. The postoperative SII index obtained in an early stage will help clinicians make an early diagnosis and provide timely interventions.

Recently, SII is considered to be more specific than CRP or erythrocyte sedimentation rate (ESR) to predict mortality in patients with cardiovascular diseases. In addition, SII is shown to be positively correlated with poor clinical outcomes of various cardiovascular diseases, including acute coronary syndrome, ST segment elevation and non-ST segment elevation myocardial infarction [21]. A higher level of SII is an independent predictor of possible future risk of cardiac death in CAD patients and the following major cardiovascular events in patients with percutaneous coronary intervention [22, 23]. Postoperative delirium is a common and serious complication after extensive surgery. Older adult patients have increased risk of developing delirium during ICU stay. Booka et al. [24] suggested that patients older than 70 years were at higher risk of developing postoperative delirium. Recently, NLR has proved to be a good predictor of delirium during hospitalization of COVID-19 patients, especially older adults, while SII could not predict delirium development in their study [25]. In our study, no significant difference in cardiovascular complication or delirium occurrence was observed using the postoperative SII cut-off value. The reason may be the low incidence of cardiovascular complications and delirium. Collectively, we suggest that the potential correlation between the SII value and cardiovascular or delirium complication occurrence still needs to be further investigated.

Our results also demonstrated that age, postoperative arterial lactate, postoperative oxygenation-index and postoperative CRP were independent predictors of SPCs. Arterial lactate is a biochemical element which is elevated in acute inflammatory phases with different etiologies. Lactate is also a strong predictor of a longer duration of the surgical procedure and prolonged hospital stay [26]. Besides, older age, lower oxygen index and elevated CRP are known factors associated with complications and poor outcomes, which is consistent with the finding of the present study. However, in our study, OR value for categorized postoperative SII is much higher than the other variables (Table 5), which means categorized postoperative SII using cutoff value is more important for predicting SPCs than age, postoperative arterial lactate, postoperative oxygenation-index and postoperative CRP.

Our research has some limitations. First, this is a retrospective and single-center cohort study and therefore selection bias and individual difference may be unavoidable. In addition, some other inflammatory markers such as PCT and IL-6 were not included in this study due to lack of some data. Finally, we did not detect SII level change over time after the first postoperative hour during the hospitalization.

In conclusion, 1-h postoperative SII, especially categorized 1-h postoperative SII using cutoff value, could be a favorable index to predict short-term occurrence of severe complications after UAS. It can help clinicians make better treatment plans in early stages owing to easy availability, objectivity, simplicity and good repeatability.

## Data Availability

The datasets generated and analyzed during the current study are available from the corresponding author on reasonable request.
